# 3D Manufacturing of Glass Microstructures Using Femtosecond Laser

**DOI:** 10.3390/mi12050499

**Published:** 2021-04-28

**Authors:** Agnė Butkutė, Linas Jonušauskas

**Affiliations:** 1 Femtika Ltd., Saulėtekio Ave. 15, LT-10224 Vilnius, Lithuania; 2 Laser Research Center, Vilnius University, Saulėtekio Ave. 10, LT-10223 Vilnius, Lithuania

**Keywords:** femtosecond laser, glass micromachining, 3D structuring

## Abstract

The rapid expansion of femtosecond (fs) laser technology brought previously unavailable capabilities to laser material processing. One of the areas which benefited the most due to these advances was the 3D processing of transparent dielectrics, namely glasses and crystals. This review is dedicated to overviewing the significant advances in the field. First, the underlying physical mechanism of material interaction with ultrashort pulses is discussed, highlighting how it can be exploited for volumetric, high-precision 3D processing. Next, three distinct transparent material modification types are introduced, fundamental differences between them are explained, possible applications are highlighted. It is shown that, due to the flexibility of fs pulse fabrication, an array of structures can be produced, starting with nanophotonic elements like integrated waveguides and photonic crystals, ending with a cm-scale microfluidic system with micro-precision integrated elements. Possible limitations to each processing regime as well as how these could be overcome are discussed. Further directions for the field development are highlighted, taking into account how it could synergize with other fs-laser-based manufacturing techniques.

## 1. Introduction

Glass and related transparent dielectrics play an important role in huge variety of applications. Through the millennia it found its way into all aspects of life, starting from construction engineering and building expanding to nanotechnology and space exploration. Glass owns its applicability to a combination of superb properties, such as being mechanically robust, transparent to the visible part of the electromagnetic spectrum, chemically inert in organic solvents and biocompatible. While glasses are not as diverse as metals [1,2] or polymers [3,4], they also can be modified in various ways [5,6,7,8] expanding their applicability. Nevertheless, ways to process glass are surprisingly limited, mostly relying on various kinds of casting [9,10] or mechanical cutting [11,12]. Due to the rapid development of science and technology, the need for more advanced and, most importantly, precise and flexible ways to produce glass structures arose. To accommodate the needs for nanotechnology various chemical deposition [13,14] or wet etching [15,16] techniques were employed. At the same time, direct [17,18] and indirect [19,20] additive 3D manufacturing of glass was demonstrated. However, in all of the highlighted processes, severe drawbacks and compromises are limiting their applicability. A more diverse and flexible approach is highly sought after.

One of the premier candidates for the best possible blend between free-form fabrication capability, flexibility, and throughput is the usage of femtosecond (fs) laser [21]. Indeed, it was already employed in a huge variety of fields [22]. The primary advantage of using ultrashort pulses is the possibility to control the thermal aspects of the light-matter interaction [23,24,25]. Furthermore, interaction can be nonlinear, meaning that materials transparent to the incident wavelength can be processed [26]. Combined, these two aspects make fs pulses extremely attractive for glass processing. Thus, this review is dedicated to highlighting how fs pulses can be employed for free-form 3D microstructuring of glass. Dielectric crystals will also be mentioned where relevant, as a lot of glass-related processing techniques can be applied for them as well. First, the physical phenomena of ultrafast light-matter interaction will be discussed, explaining how it can be tuned and localized. Then, various processing techniques based on these interactions will be explained. These include waveguide and other photonic element inscribing, zone plates, selective glass etching (SLE), and ablation. Primary attention will be given to real-life applications of shown techniques as well as connections with other manufacturing methodologies. Combined, this will provide a comprehensive outlook to the current progress of fs glass fabrication and highlight the directions where the field is headed.

## 2. Ultrafast Radiation and Material Interaction

### 2.1. The Nature of Nonlinear Radiation and Material Interaction

To give a proper background to the technologies that will be discussed further in the review, we will begin by explaining the nature of ultrafast light and material interaction. When the material is processed by using fs pulses radiation the material is affected by very high peak intensity *I* (more than GW/cm^2^). As *I* is the ratio between the laser power *P* and focused spot size with a radius of *ω*_0_, the two main ways to raise it are an increase in *P* or focus a laser beam to smaller spot size. The first requirement is fulfilled by high peak power (*P_P_*) of fs pulses (∼GW), while small spot sizes are achieved by sharp focusing—high numerical aperture (NA > 0.2) objectives or lenses. Material response to high *I* is nonlinear and conceptually is different from the material response to low-intensity radiation which is generated by non-coherent light sources. When radiation travels through dielectric material it polarizes material. When radiation intensity is not high enough material polarization linearly depends on radiation electric field *E* (1). Keeping in mind that *I*∼|*E*^2^|, when radiation creates an electric field which is stronger than 10^7^ V/m, material polarization dependency on the electric field becomes nonlinear (2).

(1) $$P\left(t\right)=\epsilon_{0}\chi^{\left(1\right)}E\left(t\right),$$

(2) $$P\left(t\right)=\varepsilon_{0}\chi^{\left(1\right)}E\left(t\right)+\varepsilon_{0}\chi^{\left(2\right)}E^{\left(2\right)}\left(t\right)+\varepsilon_{0}\chi^{\left(3\right)}E^{\left(3\right)}\left(t\right)+\ldots ,$$

here *ε*_0_ is vacuum dielectric permeability, $\chi^{\left(1\right)}$ is linear optical sensitivity, $\chi^{\left(2\right)}$—second order nonlinear optical sensitivity, $\chi^{\left(3\right)}$—third order nonlinear optical sensitivity.

Different member in nonlinear polarization equation decides different phenomenon observed in material. It also might exclude some materials from certain interaction regimes. For instance, effects that are decided by square nonlinear sensitivity, like sum and difference frequency generation, are observed only in non-central symmetrical crystals. Meanwhile, cubic nonlinear sensitivity affected phenomena are observed in all-dielectric materials. These effects are multi-photon absorption, tunneling ionization and other radiation self-interaction, such as light thread formation. It is important to note that various nonlinear light-interaction regimes can be induced simultaneously, leading to a quite complex picture of the process. Thus, when processing non-central symmetric materials a second-order interaction have to be taken into account, or how the Kerr effect might change propagation conditions when high *I* is present [27].

### 2.2. Nonlinear Effects

As it was mentioned before when the material is affected by high-intensity radiation various nonlinear light and material interaction effect appears which are governed by different order nonlinear sensitivity. One of these effects is multiphoton absorption. Multiphoton absorption is simultaneous two or more photons absorption through virtual levels from the ground to an excited state. Two-photon absorption is decided by second-order nonlinear optical sensitivity. Meanwhile, three-photon absorption is decided by third-order nonlinear optical sensitivity. Hence, higher-order multiphoton absorption is decided by higher-order nonlinear optical sensitivity respectively. Most of the time in nonlinear optics multiphoton optics is a negative effect that distorts radiation. However multiphoton absorption can be used for positive practical applications such as multiphoton spectroscopy, microscopy, and transparent material laser processing.

**Figure 1:**
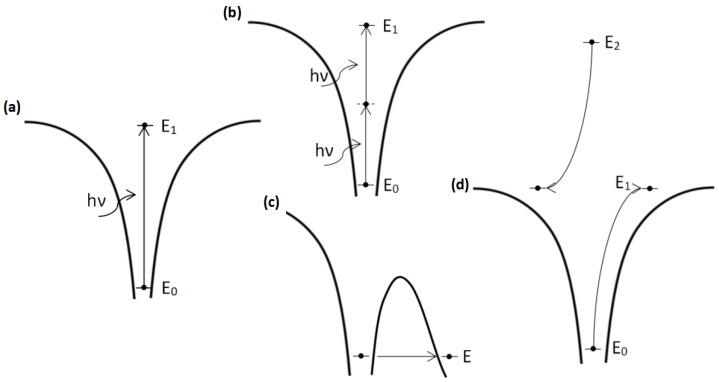
Principle schemes of main nonlinear processes: (**a**) linear ionization, (**b**) nonlinear (multi-photon) ionization, (**c**) tunneling ionization and (**d**) avalanche ionization.

In simplified terms, to induce a change in the dielectric material the electrons have to be excited from the valance band to the conductive band. Numerous processes might induce it. The most important and the most prevailing nonlinear effects when a material is illuminated with high *I* fs radiation are multiphoton ionization, tunneling ionization, and avalanche ionization. Multiphoton ionization is a process when an electron absorbs several photons simultaneously and is excited *via* virtual levels. The principal scheme of this process is shown in Figure 1b. Another nonlinear effect is tunneling ionization. Intense laser radiation can distort the potential barrier which holds electrons to material atoms. Because of the potential barrier decrease possibility of electron tunneling increases and in this way more and more electrons are excited to the conduction band. This process is depicted in Figure 1c.

Which process (multi-photon ionization or tunneling ionization) is going to dominate depends on laser *ω* and *I*. This dependency is described by Keldish parameter [28] which can be expressed as [29]:

(3) $$\gamma =\frac{\omega }{e}\sqrt{\frac{m_{e}\Delta E\varepsilon_{0}}{I}},$$

here *e*—electron charge, *m*—electron mass, *c*—speed of light, *n*—linear refraction index, *I*—laser intensity, *ϵ*_0_—vacuum dielectric penetration, Δ*E*—reserve strip spacing.

When *γ* is more than 1.5, multi-photon ionization will dominate, when *γ* is less than 1.5, tunneling ionization will dominate. Meanwhile, when the Keldish parameter is close to 1.5, transitional regime ionization occurs. In this case, multi-photon and tunneling ionization have approximately the same influence on material ionization. To sum up, tunneling ionization prevails in high *I* and low *ω* regimes. In contrast, multi-photon ionization dominates in high *I* and high *ω* cases. In laser metrical processing to reach the desired effect, it is necessary to control which effect will dominate. Fortunately, modern amplified fs-laser systems can be easily tuned to cover all the processing regimes.

Another important process that appears because of nonlinear radiation and material interaction is avalanche ionization. The principal scheme of this process is shown in Figure 1d. When radiation interacts with material electrons are released from material atoms. Continuously, irradiating material by intense electric field-free electrons is accelerated and their energy can be enough to release other electrons from neighboring atoms. In this way more and more electrons are released. Finally, the critical density of electrons in a material is reached and the material can be modified [27]. For this process to appear sufficient amount of seed electrons is required. At the same time, to achieve sufficiently high electron densities using avalanche the time for the process to appear have to be sufficiently long (>100 fs). Therefore, this process is more common when longer amplified pulses are used. Also, care should be taken, because if the process is too strong it can become uncontrollable, worsening structuring quality. Thus, care should be taken if this process is present during DLW.

### 2.3. Thermal Effects

When a material is processed with relatively long laser pulses (>10 ps) thermal effects can be observed in the material. It is the result of heat dissipation from the laser affected area which occurs while a long laser pulse is still interacting with the material in the focal point. This process takes its place around 1 ns after radiation absorption [30] and usually reduces material processing quality. When quantifying thermal effect the term Laser Affected Zone (LAZ) is introduced. It encompasses all the volume affected by the heat generated by a laser, even if it is outside of the focal point [31].

Usage of an ultrafast laser can allow control and if needed greatly reduce thermal effects caused by the light-matter interaction. Looking from the temporal perspective, interactions needed for processing occur rather fast. For instance, material ionization occurs after ∼1 ps, followed by ablation and removal after ∼100 ps [30]. Therefore, if no additional energy is introduced while subsequent thermal effects take place it can be greatly reduced. As a result in the fs pulses, the case material does not suffer from substantial thermal effects. In this way, LAZ is minimized. That is why femtosecond material laser processing sometimes is called cold material processing. Nevertheless, it is important to note that alongside pulse duration pulse repetition rate also plays important role in the thermal aspect of processing. If several pulses reach the affected zone before initial heat dissipation takes place, even fs pulses can induce material melting and noticeable LAZ. Another important factor for thermal accumulation process is the deposited energy. By maintaining the same pulse duration and pulse repetition rate and at the same time by increasing energy of each pulse the sample is affected by greater radiation dose. If the energy does not have enough time to be relaxed out of sample by increasing pulse energy we obtain higher thermal effects. Higher pulse energy shows greater affected volume around the laser focus [31] that means stronger thermal effects. While in some cases this might be detrimental, it can also be useful in some applications like laser welding. This distinction will be made through the article where it applies.

These thermal effects are especially important for glass processing. Glass is a quite fragile material that can be easily broken by treating it with stresses [32] or temperature caused volume tensions [33]. By processing glass with longer pulses or even CW laser it is very hard to avoid cracking of glass [34]. However, short pulses lasers development allows minimizing thermal effects by using shorter pulse duration [35] and process material locally without affecting surrounding volume.

## 3. Peculiarities of Glass Processing Using Fs Radiation

Light-based glass processing is not a straightforward process. Most glasses (with some exceptions like chalcogenide glasses) are transparent materials for near-UV, visible and near-IR radiation, meaning that linear interaction with such photons is negligible. As a result, one way to induce a modification in glasses is by choosing a wavelength that can be absorbed by a particular glass. Some works use such interaction for glass surface structuring based on linear glass absorption [36,37]. Such direct removal of the material by laser beam This is called laser ablation. Nevertheless, 3D processing in such cases is greatly limited due to direct absorption being a surface-bound process limiting its use for 3D structuring.

**Figure 2:**
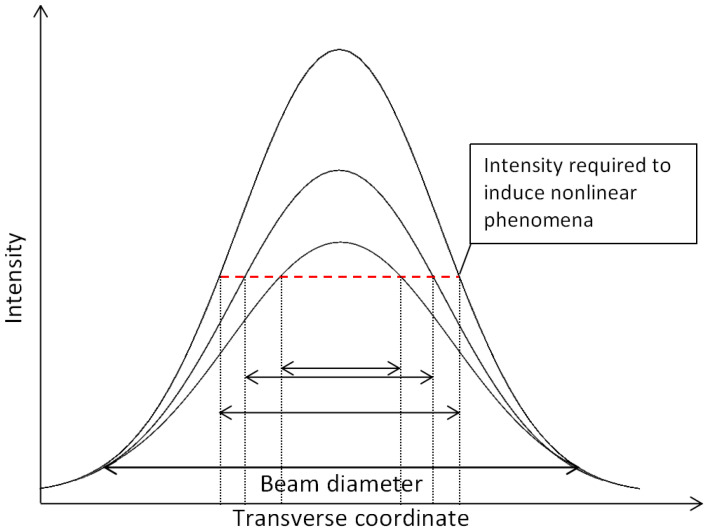
Focused laser beam spot diameter required to induce nonlinear effects dependency on radiation intensity.

Alternatively, intense and focused radiation can be used. Then interaction becomes nonlinear, resulting in melting and removal of the focal volume. For this reason, most laser-based glass 3D processing techniques are based on such processes. As we already have discussed, when radiation *I* is high enough (GW/cm^2^–PW/cm^2^) multiple photons (in most cases two) can be absorbed simultaneously during multi-photon ionisation [27]. Because high enough *I* for this interaction can only be reached by focused laser light, this creates inherent localization of the process. The scheme of this idea is shown in Figure 2. Keeping in mind, that in most cases NA in the range from 0.01 to 1.4 is used, the volume in which the modification is induced can range from a hundred nm to hundreds on µm in a transverse direction and from a few µm to several mm in a longitudinal direction. The elongated modification is an inherent feature of Gaussian beam normally used for such processing [38] while other types of voxel shapes being possible with beam structuring. Due to the nonlinear nature of these interactions sub-diffraction limited features are also possible, both directly and indirectly. Regardless, localization and selectivity of the process in the volume of glass is a critical component why fs laser is the primary tool for high precision 3D fabrication. Keeping in mind that laser can be used in a multitude of ways and not only for glass processing any laser-based fabrication process can be called direct laser writing DLW [39].

Light-glass interactions used in DLW can induce different types of modifications. These depend on laser parameters such as radiation intensity, repetition rate, pulse duration, or wavelength to name a few [40]. Of course, induced modifications depend on the material itself [40,41]. Formally, in literature, it is possible to find 3 primary stable modification types that can be induced in the glass. Nevertheless, some exceptions apply at specific cases. For instance, silver clusters can be formed in silver containing glasses, resulting in type Argentum (or type A) modification [42]. However, in this review we will concentrate at the 3 primary modification types referring to any exceptions when it applies.

**Figure 3:**
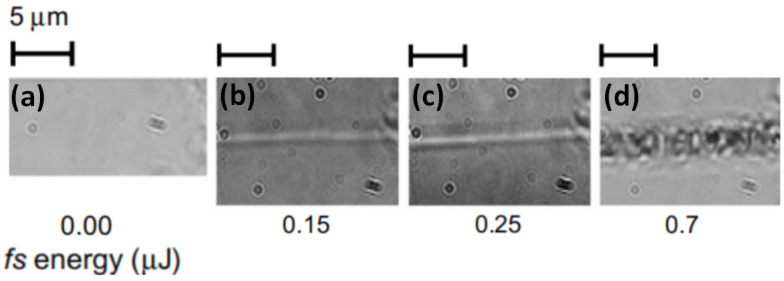
Glass modification examples. (**a**) unmodified material, (**b**,**c**) type I of glass modification or refractive index changes. (**d**)—type III modification or nano/micro-voids [40].

Type I modification is smooth material refraction index change. The change depends on various laser parameters and on the material itself. In a standard case, it is considered that such variation is mainly decided by pulse energy. An example photo of this modification can be seen in Figure 3b,c [40]. In the standard glass, the amplitude of change is in the order of 10^−3^.

**Figure 4:**
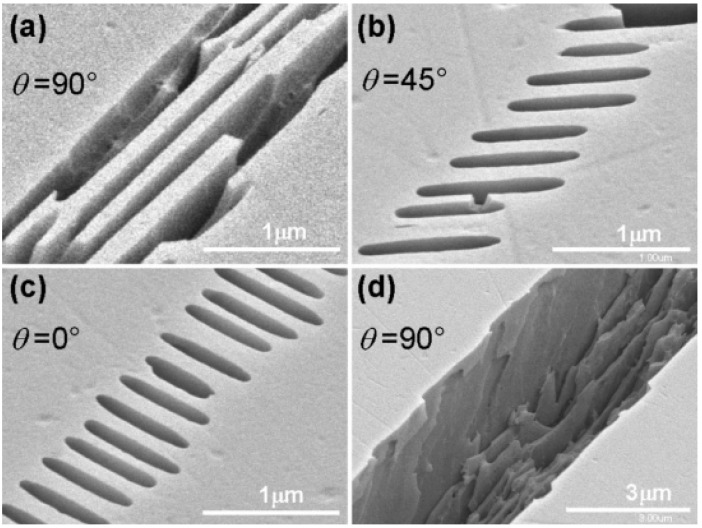
Examples of type II glass modification and visualization of nanogratings direction dependency to light polarization. (**a**) Nanogratings induced with light polarization perpendicular to scanning direction- the angle between polarization and scanning direction 90°, (**b**) 45° and (**c**) 0°. (**d**) Nanogratings modification after etching [41].

Type II of modification is periodical nanogratings in the glass. From optical standpoint, alongside changing just refractive index of the material, it also introduced birefringance. An example of nanogratings is shown in Figure 4. One of the key properties of this interaction is the tendency of nanogratings to be strongly influenced by the polarization of the incident beam. Nanograting ripple direction is perpendicular to polarization [41,43]. Moreover, the exact shape and properties of nanograting depend on other parameters as well. For instance, pulse repetition rate plays quite an important role, as it denotes the thermal aspect of the interaction [44]. The size of the ripples themselves is sub-diffraction, that is, in the range of tens of nm.

Type III modification denotes the formation of nano/micro-voids. When radiation intensity exceeds a particular value higher than needed for type II modification, micro-explosion in the volume of glass occurs and forms micro-cavities. This phenomenon is demonstrated in various works [45,46]. It is the most violent form of processing with an fs laser, leading to a relatively large modification area with several phase-change along the modified area. Particular radiation intensities to induce these types of modifications depend on the materials themselves.

## 4. Fabrication of Functional 3d Structures

There are many laser glass processing techniques based on nonlinear light-matter interactions, and by inducing different types of modification it is possible to change the method by which we perform the processing of glass. Type I modification can be used for for smooth laser refractive index modification [40] and waveguides formation in glass [47,48], type II can be used for Q-plate manufacturing, while post-processing etching step of modification is necessary for selective laser etching (SLE) [49]. Meanwhile, type III modification is used for laser ablation [35] and welding [50]. These techniques have different natures and provide various possibilities of glass modification. We will describe various laser material modification techniques in more detail later.

### 4.1. Photonic Element Manufacturing

In a broad sense, the term photonics is used to encompass all advanced light-based science areas. In the light of this section, we will use it to describe very precise structures which exploit nm-level features and/or change in refractive index to control and/or confine light in accordance to application dictated requirements [51]. In general, manipulation of thin layers of materials with different kinds of refractive index is a very widely used phenomenon. One of the primary examples of it is dielectric mirrors [52]. The Bragg law for 2D system showing where the constructive interference is the strongest reads as:

(4) $$2d\sin\theta =n_{i}\lambda ,$$

where *d* is the distance between layers, *θ*—angle of incidence, *λ*—wavelength and *n_i_*—positive integer. The most important parameter which can be controlled is the size of the features (*d*). Due to the already mention nonlinear nature of fs interaction with the transparent medium, it can be brought down to sub-diffraction level. Thus, one of the simplest examples of fs laser usage for photonic structure fabrication is the inscription of custom Bragg gratings into fibers [53]. In the simplest case, such structures are not strictly 3D. However, due to the flexibility of the technology fs laser allow forming fiber Bragg gratings (FBG) in arbitrary types of fiber, including non-photosensitized ones [54] or fibers made out of crystals [55]. At the same time, 3D capabilities are reflected by the capability to inscribe modifications in arbitrary place of the fiber, that is, not only in the core [56], or choose a specific core in multi-core fibers [57]. One of the main challenges here is focusing into the designated position within the fiber. Because the fiber shape normally is round, it defocuses the laser beam. Therefore, immersion oil [57], or flat ferules [58] might be used. This brings a lot of possibilities to the field, as it enables simple, on-demand fabrication of specialty FBGs which might be applied in various uses [53].

While FBG is a great example of a relatively simple photonic structure, 3D interactions could give substantially more diverse capabilities [51]. Therefore, the realization of 3D photonic crystals is quite an active field. Additive manufacturing was shown to be quite an attractive candidate [59]. However, in that case, the refractive index gradient between air and the polymer is around ∼0.5 which can be too sharp for some usages. At the same time, additively made 3D structures present objects on top of flat substrates, making them fragile and incompatible with anti-reflective coatings. In contrast, photonic elements embedded inside the volume of glass are robust, have a refractive index gradient of 10^−2^–10^−3^ and could be potentially coated with the anti-reflective layer. Thus, glass-based 3D photonic devices occupy a very important niche in the field.

**Figure 5:**
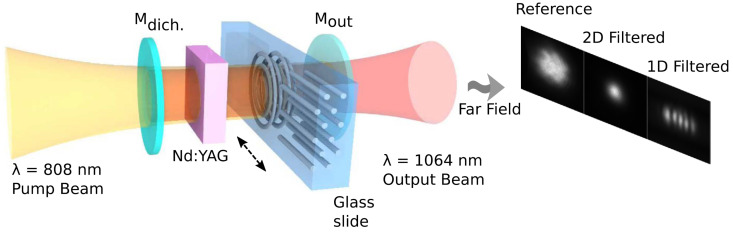
Principle of transverse mode filtering in microchip laser by application of laser-made photonic crystal embedded in a glass chip. The solution is compact and allows to achieve a high degree of control over the spatial characteristics of a laser, depending on the geometry of inserted element. Adopted from [64].

The first application is refractive index change in the glass is embedded 3D photonic crystals. It was shown that such photonic crystals can be used for spatial light filtering [60]. Indeed, the required spatial resolution and periodicity for such devices is close to the wavelength (in most cases HeNe 633 nm for simple testing) itself, making focused ultrafast radiation a go-to tool. Simple woodpile-like geometries were used first. These were produced using both laser-assisted wet etching [61] and direct refractive index modification [62]. Nevertheless, these only allow filtering along X and Y axes of the photonic crystal, limiting applicability. A solution would be to use stacked ring geometry, which then would grant this effect along all 360° in a perpendicular direction to the propagation [63]. Here possibility to integrate such a device into glass volume becomes an enabling factor. If such a device would be tried using additive manufacturing additional supports would be needed to support each ring. Furthermore, standard single-period photonic crystals only allow limiting angular filtering. As a solution to expand it asymmetrically and chirped [62] geometry was proposed and tested, again attesting to the design flexibility enabled of 3D fs fabrication. The device was then successfully tested with microchip laser filtering in transverse modes (Figure 5), enabling it to increase its brightness by 2.8–3.1 times and single transverse mode from 88 mW to 335 mW [64]. If a one-directional filter is acceptable photonic crystal writing process can be extremely sped-up by applying Bessel beams [65]. In other spatial filtering techniques, the embedded photonic crystal is one of the most compact and simple solutions available, showing clear potential wide-spread applications.

Laser-induced *n* modification can be exploited to not only shape light but also to confine and direct it. Indeed, embedded glass waveguides can be produced by using fs lasers [66]. Then, the structure has to meet the modification cross-section and Δ*n* requirements. Interestingly, waveguides can be produced by both increasing and decreasing the *n* of the material. Regarding the direction, two primary modes are exploited. At first, focusing optics move up or down in relation to the surface of the sample. This is also called longitudinal writing geometry [67,68]. The advantage is a nearly perfectly round cross-section of the modification, as well as a very uniform gradient of *n* modification to all directions. Problems, on the other hand, lies in the fact that if the sample is thick, the working distance of sharply focusing optic might be insufficient. Thus, it is more suitable for lower NA optics. Also, if non-straight modifications are needed, the uniformity and high quality of the waveguide are compromised. Alternatively, the scanning might be performed alongside the horizontal plane, that is, in a transverse manner [69,70]. Then the length and shape of the waveguide can be arbitrary. On the other hand, due to Gaussian focusing properties, the modification is elongated in the Z direction, the Δ*n* becomes highly non-uniform, especially in the Z direction. To remedy this additional elements, such as spatial light modulators (SLMs), might be needed in the optical system, complicating the setup and the process [71,72]. Alternatively, a multi-scan approach [73,74] or special light-matter interactions [75] might be exploited to make the cross-section of modification round. All of this can be combined with possibility to inscribe Bragg gratings into integrated waveguides, allowing creation of true functional 3D optical systems. It has to be stressed that it can be done in basically any kind of glass, including but not limited to borosilicate glass [76] or fused silica [77]. Thus, the needs of application dictate which of these two modes should be used. The waveguides themselves can be fabricated alone, or integrated into functional devices where they could act as a part of a sensor [78,79] or as an integrated interconnector between different types of devices [80]. This is especially attractive, as waveguide can potentially be integrated during the same technological step as the rest of the structure [78], owning to huge flexibility of fs-based processing of transparent mediums.

The elements discussed so far relied on type I modification. As a result, the designs and applicability of the produced devices were to some extent limited. To gain even greater tunability type II modification can be applied for free-form embedded optical element manufacturing. This type of fs modification allows inducing volume-embedded nanogratings which, due to orientation of sub-diffraction limited features, have distinct birefringence [81]. What is more, the directionality of the optical phenomena induced depends on the light polarization [82,83], giving huge controllability to the process. While the selection of materials in which this process is pronounced is not extremely broad [84,85,86,87], it is still sufficient to acquire a broad selection of possible optical devices. As a result it was employed to produce various optical structures, for instance diffraction gratings [88], Fresnel zone plates [89], computer-generated holograms [90,91], S-wave plates [92,93] or metamaterials [94]. Remarkable selection of possible optical elements is the result of both possibility to induce refractive index change and birefringence as well as the possibility to precisely control these modifications in 3D by using focused fs laser light. Additionally, these modifications exhibit immense thermal stability (up to 1000 °C) [95] allowing them to be considered for usage in some extreme environments. On top of that, as these elements are embedded into glass volume, their laser-induced damage threshold is also quite high (∼J/cm^2^) [96], that is, at the level of the host glass.

**Figure 6:**
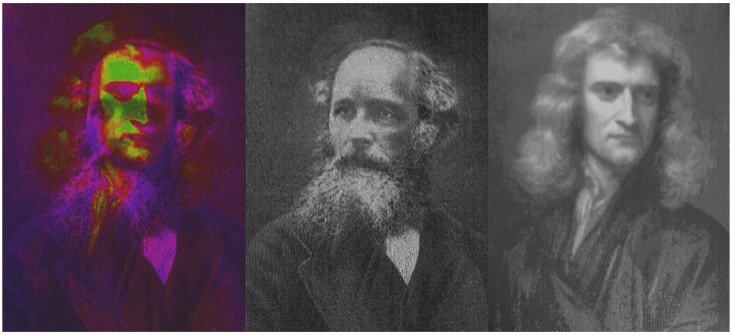
An image depicting encoding of information using type II modification induced birefringence. While both of the images seem to be written in the same volume (image on the **left**) images of Maxwell (**center**) and Newton (**right**) can be separated by exploiting imaging along both optical axes. Taken from [100].

One extremely promising application of fs-laser-based embedded photonic structure fabrication is embedded optical memory. The idea to use light for encoding and reading memory dates back to the 1980s when the laser was employed to produce and read optical discs. Over the years due to progress in optical engineering, the wavelength used for the process could be reduced, in turn increasing the data density in the disk. This is due to the fundamental need to use diffraction-limited processes for both data recording and reading. This fundamentally limits achievable data density up to ∼0.25 Gb/cm^2^ for standard systems using visible and near-UV systems [97]. However, as we discussed, fs-laser allows creating modifications not only on the surface of the material but also inside it paving the way for the volume memory [97,98]. Furthermore, single features created with fs laser can be extremely small (down to hundreds, or even tens of nm) allowing the extreme density of information [99]. Birefringence gives an additional degree of freedom as well, paving the way for true 5D memory (Figure 6) [100]. The modifications themselves have virtually unlimited lifetime [101], making them a valid candidate for extremely long-term big-data storage. Nevertheless, the modifications themselves can also be removed relatively simply if desired, granting the possibility to use the same medium to rewrite the information inside the same material volume. This can be achieved by subsequent laser exposure to the same volume [102]. Usage of crystals also allows to achieve it with relatively low temperatures (bellow 200 °C) [103] or exposure by loosely focused visible wavelength laser beam [104]. Admittedly, at the moment widespread adoption of this technology is limited due to the necessity to use expensive fs lasers and positioning. However, with the need to store up to massive amounts of information by numerous industries [105], this might become a more and more attractive solution for professional specialized extreme long-term data storage.

While the application of focused fs radiation has huge capabilities, there are some distinct limitations and hindrances native to light itself. First, if sharp focusing (NA > 0.4) is used, the polarization in the focal point has to be looked into more precisely than standard ray optics would suggest. Indeed, it is possible to show that sharply focusing fs light inside transparent medium changes *E* distribution in 3D [106,107]. Keeping in mind that the direction of type II modification heavily depends on the light polarization, this effect might have a severe influence on the result of fs processing and has to be accounted for. Furthermore, when working in the volume of a transparent medium, the laser beam is distorted due to aberrations [108]. This results in defusing effect, which changes the voxel’s size and shape (aspect ratio) in different depths of the sample [109]. The functionality of photonic crystals and waveguides heavily depends on the shape of the modification as well as the precise modification of *n*. As a result, aberrations are substantial consideration in those applications. Luckily, this issue can be solved in several ways. First, focusing optics might have some tunability, allowing to account for different depths of fabrication [110,111]. The next step is the usage of SLM [109,112]. It was shown to be a powerful tool to account for focusing deficiencies and control the aspect ratio of the feature. For instance, 1:1 to 1:1.5 aspect ratio lines can be inscribed in any depth of 1 mm sample using 0.9 NA objective [109]. Alternatively, when SLM is not used, the aspect ratio can be more than 1:25. Additionally, due to defocusing, features could not be effectively written in depths below ∼0.4 mm without significantly reoptimizing the process (i.e., increasing average laser power to compensate for defocusing. Even then, the feature aspect ratio would only increase with the depth. SLM also allows spatially shape incident beam (and, in turn, modification’s shape in 3D) for advanced structure fabrication [72,113,114]. The process is flexible enough to account even for very high *n*, like diamond’s 2.4 [115,116,117] or even anisotropic materials [118,119]. Unfortunately, SLM requires a very specialized setup, high precision in setup alignment, and advanced calculation algorithms alongside sufficient computing power [120]. On top of that standard SLMs are operating at relatively low frequencies of ∼tens of kHz limiting the possibilities for dynamic possibilities of processing, with faster systems being investigated at the moment [121]. Finally, the LIDT of SLMs is relatively low, with high-end experimental devices capable of operating at *P*∼100 W level using ultrafast lasers [122]. While solid-state SLMs might alleviate this issue [123], it remains a limiting factor. Thus, while SLMs offer a lot of interesting capabilities, their application remains limited.

### 4.2. Selective Laser Etching (SLE)

Fundamentally, type II modification of glass leads to both, nanograting formation [43] and structural changes of material [124]. The photonic applications discussed so far mainly exploited the optical phenomena of inscribed modifications. Nevertheless, appreciation of chemical and mechanical changes can lead to a very interesting prospect for further applicability. Glass, or, more precisely, Si_2_O compounds can be etched using hydrofluoric acid (HF) or Potassium Hydroxide (KOH). These etchants react with materials differently. The reaction equation of KOH and HF with one of the most popular glasses fused silica is shown in Equations (5) and (6). These reaction equations that concentrated KOH solution is beneficial over HF etchant due to least saturation behavior in elongating channel structures [125].

(5) $${\mathrm{SiO}}_{2}+2\mathrm{KOH}\to K_{2}{\mathrm{SiO}}_{3}+H_{2}O,$$

(6) $${\mathrm{SiO}}_{2}+4\mathrm{HF}\to {\mathrm{SiF}}_{4}+2H_{2}O.$$

Normally, the etch rate of Fused silica is a few μm/h, of course, it depends on etchant [126]. Nevertheless, laser-induced type II modification can greatly expedite this process. Laser radiation induces material structural variations when binding angles of the lattice change. Oxygen atoms become more reactive and more effectively interacts with the etchant [124]. Also, formed nanogratings greatly increase the effective surface area at which etchant can interact with the material. Usually, for a description of this phenomenon definition of selectivity is used. Selectivity is the ratio of etching rates of modified and unmodified material. The utilization of this process allows obtaining selectivity up to 1000 [126]. Combined with the capability to inscribe such modifications in 3D it leads to the capability to selective glass etching (SLE) [124]. The most common material used in SLE is fused silica which is almost pure amorphous SiO_2_. Fused silica SLE processing is a mostly studied process comparing to other materials. While other materials, such as borosilicate glass (BK7, Pyrex, Borofloat) [41,127,128], Foturan [129] can be used in SLE, these are rare occurrences. This is a result of lower selectivity [41] or requirement of post-processing [129,130], greatly favoring fused silica for widespread applications.

From the etchant side, HF and KOH are the most popular ones. KOH etching properties are strongly affected by KOH concentration and its temperature [131]. Usually, in the literature, we can find that 8–10 mol/L concentration KOH solution is used at 85–90 °C temperature [126,131,132]. Generally, a higher KOH concentration yield a higher etching rate. Nevertheless, a higher concentration of etchant does not guarantee better selectivity, potentially leading to contrary result [131]. This can be tied to the higher etching rate of both laser processed and unaffected areas. A similar trend can be observed with KOH temperature. When we are talking about the etching rate. It was shown, that the best selectivity is achieved at 80 °C [131]. Regarding the HF, if fast etching is desired, high (tens of %) HF concentrations should be used [133]. However, as one of the primary goals of SLE is achieving 3D structures, lower concentrations (5% or even lower) at ambient conditions are used to avoid overetching laser unexposed parts [126]. Curiously, experimentation on various conditions which might be used for SLE using HF (like temperature manipulation) is limited. Probably one of the main reasons, why more experiments are not performed which HF acid is a hazardous nature of this chemical. Subsequently, this is one of the reasons why more researcher groups prefer KOH over HF. Nevertheless, both etchants can find their applications. To get higher etching rates (up to a few hundred of μm/h) HF can be used [134]. Meanwhile, KOH should be used when high selectivity (up to 1000) is required [126], for instance producing high aspect ratio features. Of course, it is possible to combine both etchings one after another for one structure and use both etchant’s advantages. This technique is called hybrid chemical etching [132,135].

One interesting prospect of SLE is the possibility to etch crystals like sapphire (Al_2_O_3_), Yttrium Aluminum Garnet (Y_3_Al_5_O_12_ or YAG), lithium niobate (LiNbO_3_) or crystalline quartz [136,137,138,139]. It is shown that nanogratings induced in a sapphire crystal are similar to ones inscribed to fused silica [140,141,142]. However, the etching mechanism of the crystals is slightly different from amorphous materials. When focused laser beam modifies crystalline sapphire modified region becomes amorphous [143]. Then, amorphous and porous regions are etched out in aggressive etchants like concentrated (40–50%) HF at room temperature [140,141,142,144,145,146] or around 35% KOH solution heated to 85–100 °C temperature [147]. Even more exotic etchant choices were demonstrated—sapphire was etched in phosphoric and sulfuric acid mixture at 300 °C temperature [148,149]. It showed that sapphire etched in 40% HF solution has selectivity which is in order of a few thousand (up to 5000) [140,141] and this value exceeds all the results showed on fused silica. YAG crystal shows even better selectivity which is estimated to be around 10^5^ in HF acid. However, the SLE of crystals faces other problems: it is very hard to avoid cracks. Cracks are caused by modified crystalline material becoming amorphous and creating great volume tensions which lead to the crack of material [150]. Moreover, high selectivity comes from very low etching rates of unmodified material. Even modified material etching rate is very low, as little as up to 100 μm/h [137,140], for practical usage when a quite large structure is required such a low etching rate can be unacceptable.

Various laser parameters strongly affect the SLE process. A different aspect of selectivity, etched structure quality, such as surface roughness, can be decided by multiple laser parameters. Selectivity itself strongly depends on pulse energy. While sufficient pulse energy is needed to induce type II modification, the formation of LAZ can also be induced if too powerful pulses are used. LAZ affects etching properties [31]. Higher laser pulse energy with wider LAZ zones results in the wider etched zone. This effect could be for scanning up large volumes and, on contrary, this phenomena could be disadvantageous in high aspect ratio structure fabrication. Moreover, pulse duration and laser repetition rate affect LAZ and changes etching properties as well [44,151,152]. Longer pulse duration leads to stronger thermal effects. Higher pulse repetition rates prevent energy from being relaxed from the lattice. On the other hand, pulse energy not only change LAZ but also changes the size of modification and nanograting configuration itself, which leads to different selectivity induced by various pulse energies [41,43,151]. However, almost all this research leads to optimal particular parameter value which varies by changing other parameters. We can see that not individual parameters, but rather the whole parameters set is important for the SLE process. Basically, by changing pulse energy, pulse repetition rate the deposited dose is changed. Hence, a particular radiation dose is hiding under these parameter sets. Overall, it can be seen that for SLE process need to be ensured specific conditions—specific radiation dose [151] and these conditions can be altered by different thermal regime [31].

Alongside radiation parameters, scanning strategies also play an immense role in SLE effectiveness. Overall, translation velocity and pulse overlap while hatching/slicing denote the pulse overlap, which, in turn, governs accumulated radiation dose [151]. Therefore, one can consider that scanning velocity gives quite a similar effect as pulse repetition rate. As a result, selectivity dependency on scanning velocity was investigated in many works [131,151,153]. Acquired results varied, painting a more complicated picture of interaction as different sets of laser and scanning parameters were shown to work quite effectively [131,151]. Interestingly, in some works, almost no significant selectivity dependency on scanning speed was noticed [153]. The translation velocity question, in general, is very important, as it is one of the main parameters determining structuring rate, that is, what volume will be processed in a given amount of time thus better understanding of these contradicting results is a topic for further research in the field. Nevertheless, the scanning strategy also plays an important role. During the inscribing process, the specific separation between scanning lines should be chosen. Unsurprisingly, it also influences etching rate and selectivity [126,131]. One of the non-trivial observations made was that optimal Z separation value varies when different etchant is chosen [126]. Separation along a horizontal axis (i.e., slicing step) should also not exceed the voxel height [131]. Overall, due to the complex nature of the process and a huge amount of independent parameters, the exact interplay is still an object to an active investigation, with the goals of different groups distributed between trying to achieve the best selectivity, highest etching rate, lowest surface roughness or some combination of all.

It is important to not overlook the importance of polarization during the SLE process. Polarization is responsible for the direction of the nanogratings as depicted in Figure 4. Numerous works investigated selectivity dependency to laser beam polarization. When radiation polarization is perpendicular to scanning direction we get the highest etching rate [41,43]. The same applies and to selectivity because the unmodified material etching rate in all the cases remains the same [126]. Meanwhile polarization parallel to scanning direction gives the lowest etching rate [41,43]. Interestingly, circular polarization gives a good etching rate as in perpendicular polarization case [41]. This is extremely important because then the polarization might be considered invariable for any scanning strategy, heavily simplifying an already complicated process, becoming favorable for application-oriented fabrication. Nevertheless, care should be taken when considering the interplay between pulse duration and polarization. Discussed trend always appears when femtosecond laser pulses are used to write modification. Nevertheless, when ps pulses are used for SLE selectivity does not depend on radiation polarization [152]. This property comes from the nature of this process. By using femtosecond pulses it is possible to induce nanogratings modification, meanwhile, ps pulses yield modifications more reminiscent of nanocracks. These can be considered a different type of modification that is still suitable for SLE.

SLE stands in a relatively interesting place when considering piratical implementation. The process itself needs a lot of considerations in terms of laser parameters and writing strategies. On the other hand, it can be considered one of the most straightforward ways to produce 3D glass microstructures. It, as a result, possible applications were shown in microfluidics, micromechanics, optics, and photonics. In photonics, It is shown that SLE can be used for very precise photonic components fabrication. By using tightly focused laser beams it is possible to form very small (down to ∼100 nm) repeatable structures which after etching can be used as photonic components [137,148,149]. Micro optic element fabrication was also shown with SLE [130]. However, due to relatively high surface roughness after the SLE process (∼hundred nm RMS) direct fabrication of optical components is impossible. Thus, after etching some annealing or other smoothing procedure is needed.

**Figure 7:**
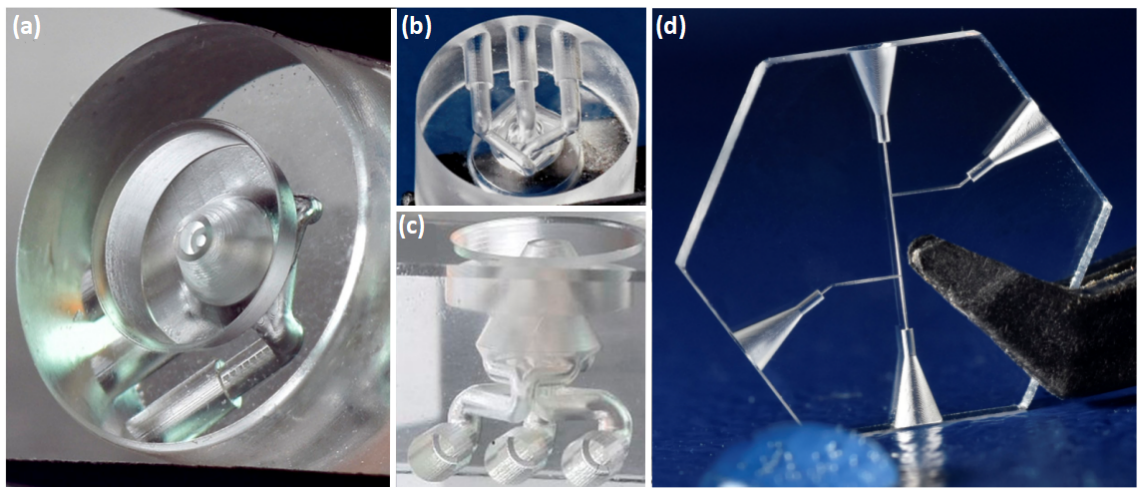
Example of microfluidic devices. (**a**–**c**) nozzle for biological applications, (**d**) glass connector for capillary electrophoresis, diameter 15 mm, thickness 2 mm [154].

**Figure 8:**
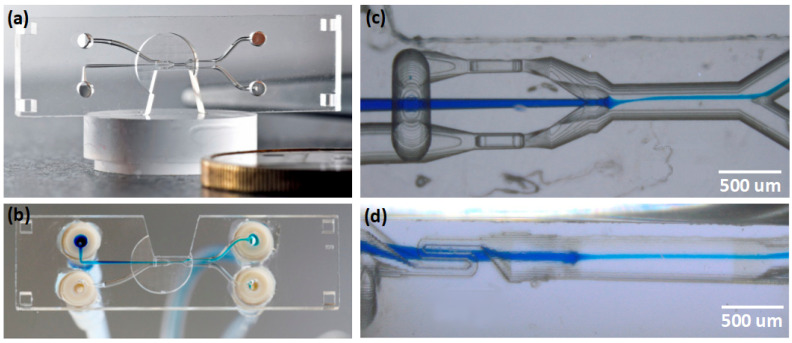
Example of microfluidic devices. (**a**,**b**) microfluidic chip created for cell sorting, (**c**,**d**) most important nodes of a chip are shown more detailed [154].

SLE excels in the field of microfluidics for the fabrication of lab-on-chip (LOC) devices. Glass is a preferred material for a lot of set applications, as it is chemically inert in organic solvents, transparent, and mechanically robust. SLE provides a relatively easy and highly controllable method for producing both surface and embedded channels. Here, SLE allows to form even complicated, curved, embedded 3D structures in glass [132,137,154,155]. It is demonstrated such channels ability to focused particles in the loops of the channels [137]. Other mentioned systems have a potential to be used in liquids filtering such as on-chip flow cytometry [137]. In the case of LOC microfluidic channel and some sort of integrated device inside it is formed [153,156]. Moreover, complicated microfluidic chains including filter and free movable integrated micro-switcher can be fabricated [132]. The mentioned device can perform fluid filtering. In the bigger channels narrower channels volume are inserted, only the smallest particles can go through the most narrow channels. Moreover, fluid flow direction can be controlled by fabricating channels with a free movable valve. Other 3D microfluidic devices which would be extremely challenging for any other technique can be fabricated [154]. To highlight some examples, Figure 7a–c show nozzle for biological applications. Figure 7d demonstrates connector for capillary electrophoresis. Figure 8 shows a microfluidic chip for cell sorting.

SLE also demonstrated huge promise in the field of micromechanics. For instance, a glass mechanical gripper can be produced and coated with metal to thermally induce movement [157,158]. A similar optical sensor can also be made [48]. The SLE-made mechanical device is coated by a metallic coat layer, which then moves when the potential changes. Information is red through the already integrated waveguide and the signal strength depends on the displacement of the structure. The beauty of this device is that it can be fabricated out of one piece of fused silica and at once it is possible to integrate waveguide and fabricate structure combining both type I and type II modifications in one fabrication step just by changing the laser parameters. Also, the device can be of arbitrary size, downscaled, and used as a component in more complex systems. Free movable micromechanical structures are also doable with SLE, such as gear systems [159]. Devices mentioned in the paragraph before conceptually are completely different from this micro-mechanical structure. In the first case, we get movement by fabricating structure with very high aspect ratio detail, so very thin details of glass become flexible. In the second case, a movable glass structure is fabricated because a slight gap is etched out between rotating and not rotating parts. Thus, by using the SLE technique, various types of movable structures can be obtained.

**Figure 9:**
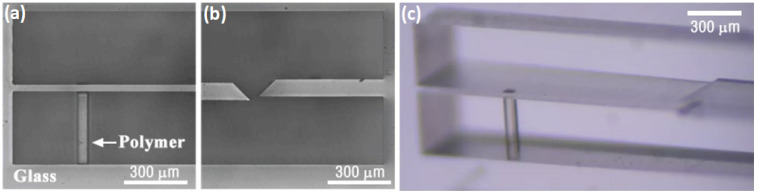
(**a**–**c**) Example of a hybrid microfluidic device [160].

Finally, one must not forget that SLE is not an isolated tool and it can be combined with other DLW techniques, including multiphoton polymerization. Such fabrication is called hybrid fs laser manufacturing. For example, chemical liquid sensing application can be realized by combining SLE and multi-photon polymerization techniques [160]. Then, the channel and integrated cantilever are made out of glass in a single fabrication step. After the SLE process, a polymeric rod is produced by using multi-photon polymerization. When the whole structure is submerged into an organic solvent glass remains inert, while the rod expands or swells, a property well documented with laser-made 3D microstructures. Thus, such a device can act as a passive actuator or a mechanical sensor. This device is shown in Figure 9. Alternatively, it demonstrates that some hybrid devices can be used for cells sorting and detecting [161,162]. In closed SLE, the channels made are integrated with polymeric lend, which allows the detection of when the cell is moving through it as well as disturbing the signal.

Overall, SLE stands in quite a peculiar place in regards to its realization and applicability. The complexity of the process means realizing it is a rather complicated task, resulting in a steep learning curve for groups that attempt to do it. For this reason, a lot of researchers tend to use the substantially simpler type III modification base ablation, which will be discussed in the next subsection. Then, to achieve the same complex structures more fabrication steps of the simpler process might be needed. It also allows avoiding potentially hazardous HF-based chemical procedures. Alternatively, advanced high precision additive manufacturing-based solutions can be employed [163,164,165], forgoing the single step SLE process in favor of several simpler fabrication procedures. However, when SLE is employed right and all the technical nuances are taken care of, it provides unmatched design freedom and flexibility in producing arbitrary shaped 3D glass structures with resolution down to µm with overall size in the cm range.

### 4.3. Free-Form Cutting of Glasses

Fundamentally, direct laser ablation is the simplest of all DLW kinds. As the material is removed from the sample without any additional technological/post-processing steps it was quickly adopted in the industry using CW or long-pulsed (ns and longer) lasers [166]. Solid-state fs lasers are gaining popularity there, as well. The draw of ultrafast lasers in laser cutting is the possibility to control the thermal aspect of the process, subsequently allowing a smaller laser affected zone and cleaner cuts [23,24,25]. This capability is further expanded by the application of burst lasers, which allow both high structuring precision as well as very high throughput. There are also virtually no limitations for the application materials, which can be organic [167,168] (including living tissue [169]) dielectric [170,171,172] or metal [173,174]. However, when considering true 3D fabrication ablation have some severe drawbacks. In comparison to 3D printing [38], complex 3D architectures, arches, and integration of structures inside structures are either difficult or, for some geometries, impossible with direct ablation. Advanced techniques allow to achieve quasi-3D structures by either filliping [174] or turning samples [175] during processing, but it requires additional fabrication steps or a more complex setup, minimizing the main advantage of ablation—simplicity. Thus, in the following discussion, we will use the term 3D rather loosely, in many cases referring to structures that can otherwise be classified as 2.5D.

Among many applications where fs ablation of glasses can be exploited, microfluidics is one of the most common. Two types of channels can be produced in that case: on the glass surface [176,177,178], and embedded inside the volume [179,180]. Surface channels are substantially simpler to produce. Also, they allow integration of additional elements using other fabrication techniques, for instance, multiphoton polymerization (MPP) [181]. However, in that case, an additional channel sealing step is needed. It can be realized in numerous ways, including direct laser welding. It will be discussed in more detail further in the article. Nevertheless, it is an additional technological step that, if possible, should be avoided. Alternatively, channels inside the bulk of the material can be produced. Indeed, fs lasers allow surprisingly very fine control of type III modifications in glass volume [182]. Despite this, if ablation is used there is no direct way for debris to leave the embedded channel if this method is used. Luckily, if the process is carried out in the water, the debris removal is expedited, resulting in the possibility to produce clean and complex channel systems [179,180], or high aspect ratio holes (for instance 4 μm wide and more than 200 μm deep [183]). By applying Bessel beam ablation it is shown even higher aspect ratio narrow channels which is around 200 nm in diameter and 20 μm in depth [184]. Alternatively, water can assist in the structuring of the volume of porous/photosensitive glass [185,186,187,188]. Then, after laser exposure, glass is heated up and solidified, embedding produced 3D system inside a material. Additional etching and annealing steps can also follow. However, then the process becomes somewhat similar in complexity (if not more difficult to perform) to SLE yielding substantially worse shape control and surface finish of the channel walls. At the same time, non-SLE methods allow using potentially simpler glasses. Regardless, apart from several demonstrations, this methodology is used sparingly.

**Figure 10:**
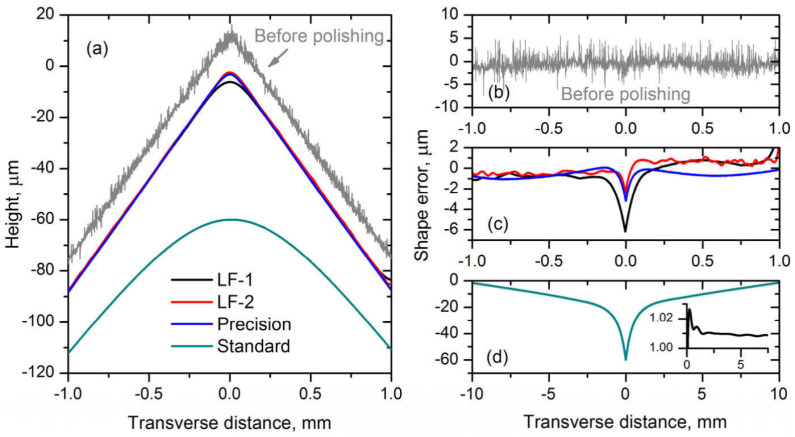
The precision of profile of the ultrafast laser-made axicon with subsequent CO_2_ polishing and comparison to commercial high-precision and standard counterparts. The un-polished and polished laser-made profiles are offset to better reveal the difference between them. Reproduced from [195].

Microoptics can also be produced using direct laser ablation. While extremely complex designs achievable using additive manufacturing are out of the question [189,190,191], ablation exceeds those methods by allowing to use of glass and achieve overall size from tens of μm to mm. Optical elements, like various type lenses [192,193,194] or axicons [195], can be produced this way. In virtually all the cases additional post-processing steps are mandatory due to the necessity to smooth the surface of the structure down to optical quality (less than *λ*/10). Annealing is the most popular option. It gently melts the surface of the optical element, allowing surface tension to increase surface smoothness without compromising the overall shape (Figure 10). This can be induced by heating the structure to a sufficient temperature (close to the melting of the material) [196], or by employing another laser such as CO_2_ [192,193,194,195]. An alternative way to achieve lenses using type III laser modification is based on the idea, of inducing very fine modifications on the surface of the material. Then the sample is submerged to the etchant, which starts to etch out the material. The modification acts as a seed, which expands over time. Due to the nature of the process, expanded areas form lens-like profiles and, after some time reach each other [197,198]. Thus, a plano concave lens array is formed with an optical grade surface finish. Overall, while ablation allows achieving free-form optics, etching provides superior shape control and a possibility to produce more complex geometries, making both approaches somewhat comparable.

Surface roughness after type III processing is rather poor (∼several μm RMS ). A laser can be employed to smooth it out. Normally, CO_2_ [199] or high power solid-state lasers [200] are employed for this task. The process is based on inducing desired thermal interactions at the surface of the sample. It can be used to polish glass parts produced using SLE [201] or ablation [202]. However, the fs laser itself can be used for this task. The primary advantage of using ultrashort pulses is the possibility to extremely precisely control thermal accumulation on the surface of the sample [203]. This can be exploited to polish some exotic materials, like Germanium [204] or yttria-stabilized zirconia [205]. Additionally, due to the controlled manner of the process fs lasers allow avoiding some of the effects associated with polishing using different laser sources or mechanical polishing. Nevertheless, the application of fs lasers in polishing remains sparse. At the same time, the advent of burst-mode capable systems brings a new level of control of thermal aspects of the interaction, potentially reducing surface roughness during processing in the first place, bringing new capabilities to the field [25].

All the processes showcased so far mostly rely on Gaussian beam focusing using standard optics. However, as discussed previously, light is a flexible tool that can be spatiotemporally manipulated. To change the spatial distribution of the laser beam, passive elements can be inserted into the path of the laser beam. In more advanced cases, it can be done using SLM, which allows the changing of a projected mask dynamically on-demand [206]. However, due to the relatively low LIDT of such elements, their usage in direct ablation might be limited. Regarding the exact applications various examples exist. For instance, the Bessel beam can allow taper-less fabrication [184]. In other words, the channel walls are nearly vertical, which is hard to achieve with sharply focused Gaussian beams. It also can be used to achieve high aspect ratio (up to 1:100) features easily [207]. Also, in the case of top-hat laser beam distribution, the cut quality can be improved [208]. Beam shaping also can be exploited to create entire patterns to be ablated [206]. Regarding the temporal properties of fs radiation, it can also be tuned to achieve desirable ablation properties [209]. Manipulation of environmental aspects should also not be overlooked. By changing the pressure during the ablation process the debris formation tendencies can be influenced, which helps with the overall quality of the sample [210]. Alternatively, additional gasses are shown to help increase ablation depth [211]. Water, which was shown to be a rather popular additive in fs glass machining, can also be used to induce spontaneous spatiotemporal light filamentation. It can be used to cut arbitrary materials, including glass, at relatively large thicknesses (1 mm and more) [212]. Thus, overall, while base fs laser ablation of glasses might be considered somewhat limited as far as 3D fabrication is concerned, some capabilities exist to enhance the result of processing. At the same time, then it somewhat complicated the processing, making it less attractive.

### 4.4. Laser Welding

Discussing 3D structure fabrication it is important to understand that in most cases a 3D structure is, in fact, multiple stacked 2D layers. Only a few processing techniques, for instance, selective laser etching, allow true 3D fabrication, yet it also relies hard on layering as it allows to minimize effects like shadowing. Sometimes the stacking is part of the process itself, like in 3D printing, while sometimes additional bonding step is required. Except for integrated free-form SLE structures, stacking glass onto glass to form 3D structures is also common, especially when parts are produced using ablation. There exist different bonding techniques such as direct, adhesive bonding, or others. Adhesive bonding is performed by using additional intermediate chemical materials [213], but it has limitations to strength as well as adds additional chemical constitutes which might be detrimental to some application. Direct bonding is performed by increasing the temperature of the samples, it starts to melt and connects with each other [214]. Thus, direct bonding by ultrafast laser welding becomes an attractive way to bond glass layers with each other forgoing most of the listed problems. Laser welding relies on type III modifications [215,216]. It can be considered one of the most advanced available bonding techniques, because of high spatial selectivity, localized material modification, and capability of completely sealing functional devices. Moreover, laser welding allows to achieve up to 95% breaking resistance of primary material [217,218]. What is more, laser welding does not require additional materials or other post-processing to be included. It is shown that laser welding can be used as a supplementary technology to strengthen other bonding techniques [219].

To weld two samples laser beam needs to be focused on the contact of those samples. Because of high radiation intensity, tunneling or multi-photon ionization occurs. Radiation is absorbed only in the focal spot where free-electron appears which mount increase because of avalanche ionization—plasma forms. Meanwhile, surrounding materials heats which enhance nonlinear which then, in turn, expedites the heating. The volume of melted material encompasses both samples which need to be bonded. Afterward, the material cools and forms a firm connection between samples [220]. By melting material some of its chemical connections are broken. In the affected material some oxygen connection remains open forming a new chemical connection between samples [221]. After measuring welded samples cross-section Raman spectra was found that welded different material samples mix in the contact [222]. Thus, this explains high welded material breaking resistance.

For a process like this optical contact is required. Optical contact appears because of inter-molecular Van der Vals bonds. If the right conditions are created for two materials surfaces to be close enough to each other, those samples start to attract each other because of mentioned forces (forces decrease proportionally to the square of the distance). Thus, in this way, we get stable optical contact [223], which does not require to be additionally supported. Even more, additional external forces applied to the samples leads to cracks in the welded seam [224]. Therefore, clean and high-quality surfaces practically became a requirement for the laser welding process. Spacing between two samples should not be larger than a few hundreds of nm, otherwise, it is hard to avoid cracks in the seam or even ablation of one of the surfaces [225]. Optical contact is one of the greatest challenges for laser welding, because often it is hard to achieve it due to surfaces imperfection of the samples, due to high surface area, or imperfections/undulations in the sample caused by other processing techniques, like ablation.

**Figure 11:**
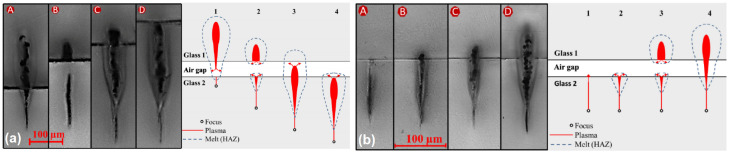
Cross-section of the welding process (**a**) welding dependency on focal position. The focal position is changed in this order from image A to D: −24.1 μm, −40.0 μm, −86.5 μm and −107.3 μm, Corresponding schemes of these process are shown from picture 1 to 4, (**b**) welding dependency on pulse energy, the pulse energy is increasing in this order from image A to D: 10.1, 11.23, 12.9 and 18.8 μJ, schemes of the corresponding process are shown in pictures 1 to 4 [220].

Advances in laser engineering positively influence the ease with which welding can be realized. It was shown how to avoid the requirement of optical contact. Optical contact is still preferred, allowing us to reach a higher breaking resistance of a welded seam than welding without optical contact can [50]. However, for simpler, less challenging applications even simplified welding is sufficient. At this moment, it ihas been shown that laser welding of samples with gaps between each other up to 3 μm [220]. There a few factor with allows to walk around this requirement. First of all, the gaps which could be efficiently filled depends on the amount of material that is brought to the gap. This amount of material can be evaluated as the size of a welding seam. Seam size directly depends on laser radiation and scanning parameters (pulse energy, pulse repetition rate or scanning velocity, focus position in the Z direction in respect of the contact of samples) used for the welding process or even it could depend on material thermal conductivity. Thus, it possible to lower the high-quality contact requirement by increasing the size of the seam. Welded seam size can be increased in a few ways. The usage of high repetition rate (hundreds of kHz) radiation leads to a greater size of a seam [225]. The same applies to higher pulse energies [50,218,221]. This dependency is schematically shown in Figure 11b. By bringing more materials to the contact of the sample, it not only provides the possibility to weld samples with larger gaps but also affects the breaking resistance of welded samples. A larger welding seam will lead to higher breaking resistance [217]. Care should be taken, however, because of the possibilities of breaking material due to high internal tension instead of material welding [226,227].

It is important to understand how scanning properties affect the size of the seam. Lower scanning velocity will lead to higher pulse overlapping resulting in more energy being delivered to each point. As a consequence larger welding seam and higher breaking resistance are obtained [221,228]. What is more, the gap between separate seams, or hatching step, affects samples breaking resistance significantly [50,218]. It is quite intuitive that a larger amount of seams in the same sample allow the obtaining of a greater breaking resistance. On the other hand, welding sample by separate seams brings its own benefits to this process. Distinguished seams give a possibility to contact materials with different thermal expanding coefficients. Because the connection is very local, the spacing between seam compensates for the fact that the two materials expand differently. Unsurprisingly, the focal spot position in the Z direction in respect of gap position is also a critical parameter in laser welding. The way this parameter influences the process is shown in Figure 11a. It can be observed that, in laser welding, for the most effective process the focus of the radiation should be a little bit (∼100 μm) below the contact of the samples [220]. In this way, melted material comes up to the contact and fills it in the most efficient way. This tactic allows us to melt more material and fill the gap between samples with the greatest amount of material.

The advent of burst lasers have also been experimented with in welding. It allows improvement of the welding process in many aspects. Burst-welds have higher breaking resistance than that achieved by only welding in a high repetition rate regime [229]. Moreover, usage of burst enables us to weld materials with greater spacing in the contact between samples [50]. Another interesting approach is laser welding with Bessel beams [222]. One of the unique Bessel beams property is long focal length. Its relay length can up to a few hundred μm. Thus, in this way we do not need to have accurate information about the position of the contact of materials.

Laser welding can be applied in many functional device fabrication. It can be applied in such areas as electronics [230], microfluidics [213]. This technology could be extremely useful in electronics when the whole chip cannot be processed in other bonding techniques and local laser welding means that important parts of the chip can be left unaffected. Ultrafast welding is also advantageous, as it allows to easily bond glasses with other materials like metals or polymers. However, due to high technical requirements for both laser systems and surfaces of bonded samples, the advance of this technique is so far slow, with most demonstrations being done only in academia with proof-of-concept work.

## 5. Conclusions

The field fs laser-based processing of glasses and transparent dielectrics is wast. In this review, we have shown that:

1. Due to the nonlinear nature of fs pulses-based light-matter interaction, transparent to the used wavelength dielectrics can be processed, including glasses and crystals. This opens doors for true 3D processing, including inside the volume of the material. Due to interaction peculiarities, three distant types of modifications can be formed. Type I results in a localized smooth refractive index change in the range of Δ*n* = 10^3^. This can be used to create embedded waveguides, Bragg gratings, and other integrated photonic structures.
2. Type II modification is created with higher pulse energy, which manifests as embedded, sub-diffraction-limited nanograting. Characteristics of such ripples depend on pulse energy and polarization. They also have pronounced birefringence. These can be used to produce various optical and photonic elements. If put into an etching solution, such as HF or KOH, these also etch out from tens to thousands of times faster, enabling selective 3D glass etching.
3. Due to the nature of type III modification, that is, ablation can be used for applications demanding less precision but higher precision, such as microfluidics. Application in optics is also possible, but additional post-processing is needed. Overall, this processing type partially sacrifices true 3D processing capability in exchange for being a lot simpler and faster.
4. As fs lasers allow us to precisely control thermal effects during processing, they can be employed for polishing and welding. In the latter case, it allows the joining of glasses of the same and different types, as well as glasses with plastics or metals, making it one of the most versatile tools available for such applications.

Overall, the key advantage of the fs laser is flexibility. Such processing can allow achievement of the same 3D structure using very different methodologies, allowing us to choose a process on a case-by-case basis. With this flexibility, one can expect the field of fs pulse-based glass processing to continue to grow rapidly in the near future, with both wider applications of existing methodologies and the creation of new ones.
